# Unraveling the Enigma of Moderate Aortic Stenosis: Challenges and Future Prospects

**DOI:** 10.3390/jcm13123478

**Published:** 2024-06-14

**Authors:** Gloria Santangelo, Gabriele Tumminello, Lucia Barbieri, Giulio Pio Federico Mallardi, Andrea Faggiano, Silvia Moscardelli, Andrea Rossi, Fabiana Cozza, Stefano Carugo, Pompilio Faggiano

**Affiliations:** 1 Department of Cardio-Thoracic-Vascular Diseases, Foundation IRCCS Ca’Granda Ospedale Maggiore Policlinico, 20122 Milan, Italy; gloria.santangelo@policlinico.mi.it (G.S.); gabriele.tumminello@policlinico.mi.it (G.T.); lucia.barbieri@policlinico.mi.it (L.B.); giulio.mallardi95@gmail.com (G.P.F.M.); andreafaggiano95@gmail.com (A.F.); stefano.carugo@policlinico.mi.it (S.C.); 2 Department of Clinical Sciences and Community Health, University of Milan, 20122 Milan, Italy; 3 Division of Cardiology, Department of Health Sciences, San Paolo Hospital, University of Milan, 20122 Milan, Italy; silvia@moscardelli.it; 4 Ospedale Magalini, 37069 Villafranca, Italy; andrea9rossi@gmail.com; 5 Cardiothoracic Department Unit, Fondazione Poliambulanza, 25124 Brescia, Italy; fabianacozzamd@gmail.com

**Keywords:** moderate aortic valve stenosis, left ventricular disfunction, discordant moderate aortic stenosis, aortic valve replacement, iPCSK9

## Abstract

According to current guidelines, only clinical surveillance is recommended for patients with moderate aortic valve stenosis (AS), while aortic valve replacement may be considered in patients undergoing surgery for other indications. Recent studies have shown that moderate AS is associated with a high risk of adverse cardiovascular events, including death, especially in patients with left ventricular dysfunction. In this context, multimodality imaging can help to improve the accuracy of moderate AS diagnosis and to assess left ventricular remodeling response. This review discusses the natural history of this valve disease and the role of multimodality imaging in the diagnostic process, summarizes current evidence on the medical and non-medical management, and highlights ongoing trials on valve replacement.

## 1. Introduction

Aortic valve stenosis (AS) is a disease characterized by the thickening and calcification of the aortic cusps with consequent narrowing of the aortic orifice. The severity of AS is typically classified as mild, moderate, or severe, depending on the extent of narrowing. This is typically determined by measuring the peak velocity across the valve using transthoracic Doppler echocardiographic, thereby obtaining the peak pressure gradient between aorta and left ventricle (LV), the mean pressure gradient, and the aortic valve area (AVA) using continuity equation [1].

According to current guidelines [2], a conservative approach of watchful waiting is recommended for moderate AS, irrespective of patients’ risk profiles and symptoms.

Nevertheless, three interconnected clinical concepts suggest that an early interventional approach could be beneficial in this setting. First, recent observational studies have revealed a heightened risk of adverse cardiovascular events associated with moderate aortic stenosis. Second, there has been an undeniable improvement in periprocedural, perioperative, and long-term clinical outcomes, including complication rates and mortality, following surgical and transcatheter aortic valve replacement (SAVR and TAVR), which challenges the traditional risk/benefit paradigm [3]. Finally, the progression in the severity grade of stenosis is both inexorable and predictable. 

These considerations are particularly valuable in patients with concomitant LV dysfunction, where moderate aortic stenosis may have a worse clinical and hemodynamic impact. Especially in this setting, it is reasonable to consider a case-by-case evaluation by a Heart Team, taking into account variables like the “near-severe” valvular disease, age, frailty, comorbidities, operative risk. and the most suitable intervention option available [4].

In this review, we summarize the current state of knowledge on the epidemiology, rate of progression, and outcome of moderate AS. We emphasize the crucial necessity for an accurate diagnostic pathway, especially in case of discordance between AVA and pressure gradient, a common scenario in patients with concomitant LV systolic dysfunction. Finally, we discuss the current therapeutic options available, considering ongoing trials, which may soon bring about a shift in the management of moderate AS.

## 2. Epidemiology

Calcific aortic stenosis (AS) is the most common valvular disease in Western countries [5]. Moreover, as life expectancy increases worldwide, particularly in developed countries, the burden of AS is expected to escalate along with the need for intervention [6]. The prevalence and incidence of moderate AS are indeed less well understood compared to severe aortic valve disease. This discrepancy arises because severe AS has been historically associated with higher morbidity and mortality, thereby attracting more clinical and research focus. However, large observational studies have demonstrated a high overall mortality associated with moderate AS.

A meta-analysis estimates that about 8% of the elderly population (>75 years) suffers from mild or moderate AS, compared to 3.4% with severe AS [7]. Recently, in a study of all patients who underwent an echocardiogram at a single medical center, it was found that 2.4% of adults had moderate AS (mean age 78) [8].

Moreover, a study by Strange et al., which involved 3315 patients with moderate AS across 12 sites in Australia, revealed that the 5-year mortality rate for patients with moderate AS was 56%, nearly comparable to the 67% mortality rate observed in patients with severe AS, even after adjusting for LV dysfunction [9].

### Risk Factor and Comorbidities

In the Canadian CANHEART study, hypertension, diabetes, and dyslipidemia were identified as the most significant predictors of AS [10]. Additionally, in the Copenhagen General Population Study, having a body mass index (BMI) of 35.0 kg/m^2^ or higher was identified as an important risk factor [11]. In the Swedish General Population Study, higher BMI, obesity, cholesterol, hypertension, atrial fibrillation, smoking, and family history for stroke were associated with AS [12].

Some studies have reported an increased prevalence of cancer in patients with AS [13,14], likely due to sharing significant risk factor such as the age [15]. Additionally, the inflammatory state associated with malignancies has the potential to exacerbate the progression of AS through the development and advancement of atherosclerosis.

Many studies have demonstrated the pathological connection between Lp(a) and AS, especially detecting a link between higher Lp(a) and faster progression of AS leading to a greater need for aortic valve replacement (AVR) [16]. However, according to a recent study, Lp(a) is involved in the onset of aortic valve calcification rather than its progression [17]. The development of AS begins with calcification of the valve cusps, leading to remodeling of the valve. The link between Lp(a) and AVS is mainly due to the ability of Lp(a) to bind to the endothelial surface and infiltrate the internal layers of the aortic valve [18].

Another disease associated with AS is cardiac amyloidosis (CA), which usually co-occurs with age. This association is not uncommon in the elderly population, yet probably underestimated despite the recent implementation of a specific algorithm to detect CA. In a study among transthyretin (TTR) CA patients, the prevalence of moderate to severe AS was 27% [19]. Pesko et al. [20] aimed to compare the survival rates of patients with both CA and AS, in particular moderate and severe AS, with those with only CA and no AS. The results revealed that there was no statistically significant difference in two-year mortality between the two groups (37% and 33% respectively). It is noteworthy that half of the patients in the first group underwent AVR. These results highlight the importance of detecting AS in patients with CA, as early treatment can lead to comparable prognosis to patients without valvular disease. Moreover, in the context of CA, the LV becomes more pre-load dependent due to ventricle stiffness. Therefore, afterload reduction could potentially benefit patients with moderate AS with CA. Further studies are needed to explore this possibility.

## 3. Diagnosis

Transthoracic echocardiography remains the primary noninvasive method in the evaluation and grading of AS. Current American and European recommendations for grading severity of AS are based on three main hemodynamic parameters: AVA, aortic peak jet velocity, and mean transvalvular pressure gradient [21]. AVA is calculated by using the continuity equation, which is based on the concept that the stroke volume (SV) ejected through the LV outflow tract (LVOT) all passes through the stenotic orifice, and thus SV at valve orifice level is equal to the LVOT SV [1]. Pressure difference between the LV and aorta in systole, or transvalvular aortic gradient, is another standard measure of stenosis severity. Transaortic pressure gradient is calculated from peak jet velocity using the simplified Bernoulli equation. The mean gradient is calculated by averaging the instantaneous gradients over the ejection period, a function included in currently available clinical instrument measurement packages using the traced velocity curve [1]. According to European Society of Cardiology/European Association for Cardio-Thoracic Surgery [ESC/EACTS] guidelines, moderate AS is defined by an AVA between 1.0 to 1.5 cm^2^, a mean pressure gradient between 20 and 40 mmHg and a peak velocity of aortic jet between 3 and 4 m/s [22] (see Table 1).

### 3.1. Pitfalls

**Figure 1:**
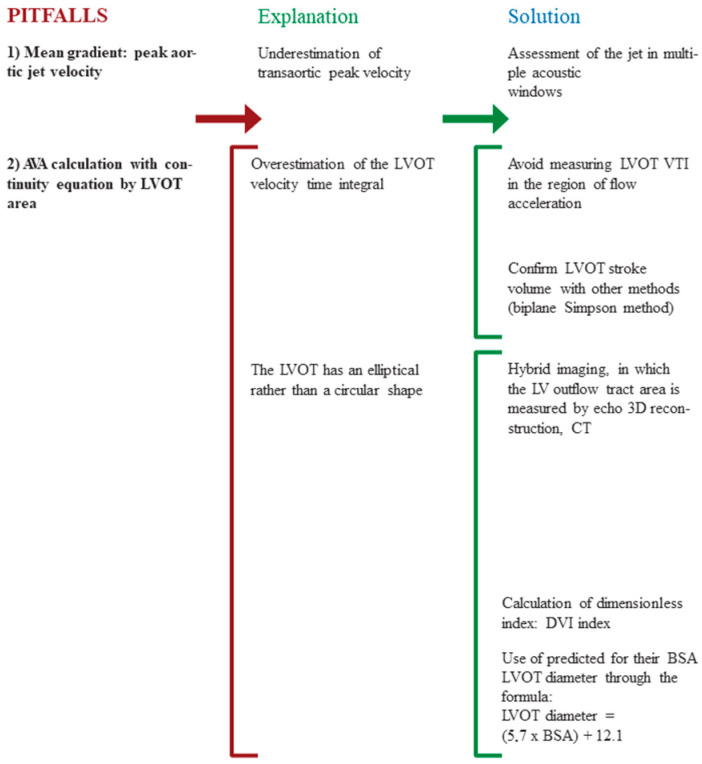
Echocardiographic pitfalls in grading aortic stenosis. BSA: body surface area; CT: computed tomography; LVOT: left ventricular outflow tract; TTE: transthoracic echocardiography; VTI: velocity–time integral.

Measurement errors and difficulties can contribute to discordant echo/Doppler assessment of AS severity. The most common errors include underestimation of transaortic peak velocity, overestimation of the LVOT velocity time integral [23], and errors in measuring the true cross-sectional area of the LVOT. The use of hybrid imaging, through echo 3D reconstruction, computed tomography (CT), or cardiac magnetic resonance (CMR), may enhance the accuracy of grading with concordant parameters. For a precise evaluation, especially in cases of discrepancy between symptoms and AS severity, the usefulness of all the thoracic views (including the right parasternal, suprasternal view, and off-axis) and of the “Pedoff” probe should not be forgotten to avoid an incorrect underestimation of severe AS as moderate [24]. Another valuable option, when transesophageal echocardiography is performed, is to directly measure AVA by 3D planimetry reconstruction. In case of discordant measures, the calculation of the dimensionless index (DI), which represents the ratio of LVOT time velocity integral to the aortic valve time velocity integral, eliminates the measurement of LVOT diameter in reducing errors derived from the AVA calculations (DI < 0.25 truly defines severe AS; DI between 0.25 and 0.5 defines moderate AS, and DI > 0.5 defines mild aortic stenosis). The most common echocardiographic measurement errors and pitfalls in grading AS severity are summarized in Figure 1.

### 3.2. Discordant Moderate AS

Even after exclusion of measurements errors, approximately 20% to 40% of patients with moderate AS still exhibit discordant measures of severity [21]. This is often associated with the presence of a low-flow state (SV index < 35 mL/m^2^ or mean transvalvular flow rate < 210 mL/s).

In the context of discordant moderate AS we can identify three different main clinical scenarios:

1. Severe AS by AVA and moderate AS by pressure gradient (AVA < 1 cm^2^, mean pressure gradient < 40 mmHg) in the presence of low-flow state (SV index < 35 mL/m^2^): low-flow low gradient AS; subclassified in “classical” when LVEF is <50% or “paradoxical” when LVEF is >50%.
2. Moderate AS by AVA and severe by high pressure gradient (AVA 1–1.5 cm^2^ and mean pressure gradient > 40 mmHg).
3. Moderate AS by AVA and mild AS by pressure gradient (AVA 1–1.5 cm^2^, mean pressure gradient < 20 mmHg).

In the first scenario, a patient with severe AS according to AVA measure and reduced LVEF, a low dose (up to 20 microg/kg/min) dobutamine stress echo (DS)E is recommended in order to differentiate low-flow low-gradient true severe AS (TS AS) from pseudo-severe AS (i.e., moderate, PS AS). Indeed, during DSE, if the patient demonstrates evidence of flow-reserve (FR, i.e., a percentage increase in SV of ≥20%), it is possible to reclassify the patient as having PS AS if the AVA increases to >1 cm^2^ while maintaining the mean pressure gradient < 40 mmHg. Conversely, in the presence of FR, AS is defined as TS if the AVA remains <1 cm^2^ and the mean pressure gradient increases above 40 mmHg during DSE. However, traditionally, this evaluation to discriminate TS from PS AS is limited by the dependence on the magnitude of flow increase achieved during DSE, which is highly variable between patients. To enable an accurate comparison of results between patients, the TOPAS study has proposed a new stenotic index that is standardized for flow: the projected AVA at normal flow rate of 250 mLs (which is the median value of the normal flow range) [25]. The proposed formula for the projected AVA is as follows:

Projected AVA = AVARest + [(AVA peak − AVA rest)/(Qpeak − Qrest)] × [250 − QRest]

Here, AVARest and QRest represent the AVA and mean transvalvular flow rate (stroke volume/LV ejection time) at rest, while AVA peak and Qpeak represent the AVA and mean transvalvular flow rate at peak stress during DSE, respectively. The main limitation of this index is that it remains flow-dependent, with a minimum of 15% increase in flow rate required for conclusive results. Therefore, in patients with inconclusive DSE or in those in whom DSE is contraindicated, a CT quantification of aortic valve calcification (CT-AVC) stands out as extremely useful in the assessment of AS severity, especially in cases of paradoxical low-flow low-gradient.

The second scenario (moderate AS by AVA and severe by high pressure gradient (AVA 1–1.5 cm^2^ and mean pressure gradient > 40 mmHg) has been recently described as discordant high gradient AS (DHG-AS). Firstly, this group excludes cases of reversible conditions of high flow (which can overestimate the gradient) such as anemia, hyperthyroidism, arterio-venous fistulae, and hypertrophic obstructive cardiomyopathy. In these patients, reevaluation is recommended when SV and cardiac output are within normal limits. Following the exclusion of conditions associated with high flow, patients identified as having true DHG-AS exhibit a less favorable prognosis when compared to individuals with concordant moderate AS, with survival rates that closely resemble those observed in cases of true severe AS [26]. Recent studies [27,28] have indicated that DHG-AS is not uncommon, with a prevalence of 10% among patients with mean PG exceeding 40 mmHg. These patients typically present with larger body surface areas, LVOT diameters, LVEF, SVi, and higher rates of aortic regurgitation, suggesting that these anatomical factors result in increased opening of the aortic valve leading to the discordant value. Furthermore, the incidence of bicuspid aortic valve (BAV) is higher in DHG-AS patients compared to those with concordant high-gradient AS. Indeed, BAV is associated with “horizontal-oval”-shaped LVOTs, potentially distorting AVA estimation using the continuity equation, leading to the discordant value. Despite their likely poor prognosis, this subset of AS severity remains understudied. Misinterpretation of DHG-AS may lead to delayed referral for AVR and increased patient morbidity and mortality.

**Figure 2:**
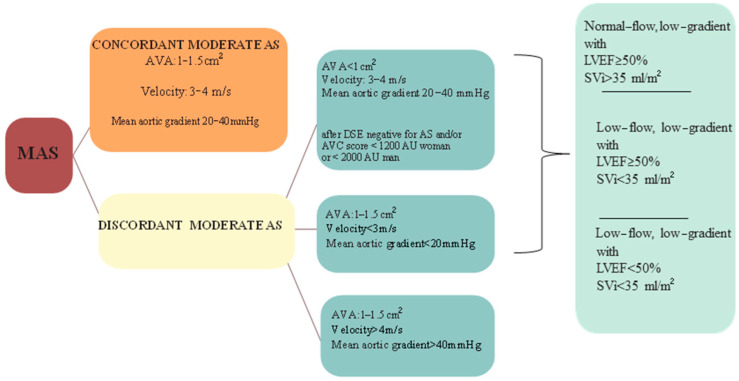
Proposed classification of moderate aortic stenosis. AS: aortic stenosis. AVA: aortic valve area. AVAi: aortic valve area index to body surface area. AVC: aortic valve calcium. AU: Agatston units. DSE: dobutamine stress echocardiogram. LVEF: Left ventricular ejection fraction. MAS: moderate aortic stenosis. SVi: stroke volume index to body surface area.

The latter scenario (moderate AS by AVA and mild AS by pressure gradient) was recently analyzed in a study of Stassen et al. [29] (see Figure 2). The authors found that patients with discordant moderate AS (moderate AS by AVA and mild AS by pressure gradient) exhibited significantly higher mortality rates during the 1-, 3-, and 5-year follow-up periods (17%, 32%, and 47%, respectively) when compared with patients with concordant moderate AS (10%, 24%, and 36%, respectively) and were less likely to undergo AVR (17% vs. 40%). The worse prognosis, as expected, was attributed to those individuals with a low-flow state, regardless of left ventricular ejection fraction (LVEF). In fact, the subgroup of patients with moderate AS by aortic valve area and mild AS by pressure gradient with normal flow conditions had incidence of outcomes comparable to those with concordant moderate AS.

## 4. Progression and Prognosis

Regardless of its initial severity, AS progresses over time, and many epidemiological studies have attempted to better understand its natural history. Grasping the natural course of AS, from valve sclerosis to severe stenosis, is crucial for making informed clinical decisions. Recent data indicate that around 40% of patients with moderate AS develop severe stenosis within approximately 2.5–3 years [30]. Hence, it is not surprising that the data unequivocally indicate poor long-term survival in patients with moderate AS. In the general population, the presence of moderate AS has been shown to be associated with a 2.3-fold increase in mortality compared with the absence of AS and a 1.3-fold mortality increase compared with mild AS [9]. Data from the Heart Valve Clinic International Database revealed a rate of 78% mortality in moderate AS patients during an 8-year follow-up [31].

In the early 1990s, initial reports regarding the rate of AS progression emerged, revealing an average annual increase in peak velocity of 0.4 m/s, peak pressure gradient of 15 mmHg/year, mean pressure gradient of 7 mm Hg, and an average reduction in aortic area of approximately 0.1 cm^2^/year [32,33,34]. Additionally, at a 5-year follow-up, around 65% of patients with aortic valve sclerosis developed overt AS: 44% exhibited mild, 14% moderate, and 8% severe AS. There is obviously a marked individual variability, with more rapid progression in older patients and in patients with more severe leaflet calcification. Recognized predictors of rapid AS progression include initial degree > mild of AS, associated LV hypertrophy, New York Heart Association classification of heart failure (HF) class III or IV, AVC, and certain demographic features such as male gender or Caucasian race [3,35,36]. The more severe impact in males could be attributed to the fact that, whereas women tend to develop more fibrotic valve remodeling, men experience greater aortic valvular calcifications, which accelerate disease progression [37]. Instead, faster disease progression in Caucasians compared to African Americans (decrease in AVA cm^2^/year 0.075 ± 0.005 vs. 0.062 ± 0.004, *p* = 0.03) is likely due to the higher prevalence of bicuspid aortic valve in Caucasians [35].

Regarding baseline anatomic valve features, Willner et al. [38], in a meta-analysis of 5450 patients from 24 studies found that AS progression rate was strongly associated with baseline mean gradient, peak velocity, and AVC, but not with peak gradient or AVA. Recently, Cho et al. [39] confirmed this by identifying two patient group with moderate AS: one with slow progression and one with rapid progression. A higher initial mean pressure gradient (=24 mmHg) was associated with rapid AS progression and higher AVR rates highlighting the predictive value of the baseline mean pressure gradient.

More recently, advancements in multimodal valve imaging technology have provided fresh insights into predicting disease progression and prognosis. Seo et al. [30] tested Doppler-derived pressure increase per unit of time (dP/dt), first described by Mason et al. in 1964 as a novel echocardiographic parameter to forecast the progression from moderate to severe AS [40]. An increased dP/dt indicates higher flow acceleration and progressive aortic valve damage, raising the risk of deterioration [41]. The study found that a dP/dt over 600 mmHg/s is associated with the progression to severe AS in patients with moderate disease [30,42]. On the other hand, positron emission tomography (PET)/CT, particularly 18F-NaF PET/CT, stands out as the exclusive diagnostic tool capable of evaluating the activity of valvular inflammation and calcification in vivo. Thus, it comes as no surprise that a recent study has identified the significant utility of 18F-NaF PET/CT in predicting the hemodynamic progression of calcific AS [43]. Furthermore, the application of artificial intelligence could prove extremely valuable in this context. Recently, an artificial intelligence algorithm was developed using a database of 1,163,923 echocardiographic reports, capable of predicting the rate of progression from moderate to severe AS. This algorithm’s predictions estimated that approximately 17.2% and 41.3% of the population would progress to severe AS at 1 and 2 years, respectively. Moreover, this model can identify subpopulations of patients more likely to progress to severe AS. It could be of great assistance to physicians in identifying and prioritizing patients who require careful follow-up after the diagnosis of moderate AS, potentially more closely than recommended by guidelines [44]. Imaging is not the only valuable tool for understanding disease progression and prognostic stratification in moderate AS. Circulating biomarkers, particularly N-terminal prohormone of brain natriuretic peptide (NT-proBNP), play a crucial role. Recent evidence shows that individuals with non-severe AS and NT-proBNP levels within the reference range, maintained over one year, have a low clinical risk in the subsequent two years. Conversely, the combination of a 1-year increase in NT-proBNP and a 1.5-fold rise from baseline is associated with the highest clinical event rates in patients with mild (hazard ratio: 8.12) and moderate AS (hazard ratio: 4.05) [45]. Moreover, combining the assessment of NT-proBNP with Galectin-3 measurement could enhance its prognostic power in predicting both the progression of valvular disease and clinical events [46].

Lastly, it crucial to note that moderate AS, even when asymptomatic, can lead to changes in myocardial structure affecting prognosis. CMR studies have documented focal irreversible fibrosis and scar formation [47]. In this setting, LV fibrosis can be categorized into reactive interstitial fibrosis, reversible following AVR, and focal replacement fibrosis, reflecting more advanced disease and typically irreversible [46]. Possible explanations for fibrosis include oxygen supply–demand mismatch in hypertrophy and ischemia due to microvascular coronary artery disease [48]. Stassen et al. showed the prognostic value of LV remodeling patterns in moderate AS, with concentric hypertrophy being associated with all-cause mortality risk [49]. Assessment of the LV remodeling patterns may identify patients at higher risk of adverse events, warranting closer surveillance, and possibly earlier intervention [49]. Even LV concentric remodeling (not overt hypertrophy), along with diastolic dysfunction, is associated with worse outcomes [46]. Similarly, speckle tracking LV global longitudinal strain (GLS) may help asses early myocardial dysfunction with impaired GLS, which mostly depends on the presence of mid-wall fibrosis [48], indicating poor survival outcomes even after AVR [50]. In light of the above-mentioned evidence that AS progressively damages the heart, Genereux et al. proposed a new staging system for AS based on extra-aortic valve cardiac damage [51] showing significant decrements in survival by stage. This staging system, successively implemented with GLS, diastolic dysfunction, and flow-state by Tastet et al., provides incremental prognostic value [51].

Table 2 shows six of the most representative studies of last decades on the prognosis of patients with moderate AS.

### Moderate Aortic Stenosis and Left Ventricular Disfunction

The coexistence of AS and LV dysfunction is common, especially in elderly patients. Indeed, approximately 10% of patients with AS have HF with reduced ejection fraction (HFrEF) [55]. Data from the Duke echocardiographic database revealed that 1.2% of patients would meet the criteria for moderate or severe AS with reduced LVEF, including 0.8% with moderate AS and 0.4% with severe AS [56]. An echo-lab performing around 5000 echocardiograms annually diagnoses moderate AS with reduced LVEF in 40 patients per year, highlighting a significant clinical burden [57]. This condition is likely underestimated in the general population and expected to rise as the population ages [58]. Common wisdom has traditionally suggested that patients with moderate AS do not have an hemodynamically significant valve obstruction capable of causing symptoms or functional impairment [59]. Consequently, AVR in patients without hemodynamically significant obstruction has been considered to carry unnecessary risks without clear anticipated benefits.

However, patients with moderate AS and impaired LVEF have a particularly poor outcome, with over 60% experiencing HF hospitalization or death within a 4-year follow-up [60]. In a large contemporary analysis by Généreux et al., involving almost 600,000 patients, untreated moderate AS was associated with an adjusted 4-year mortality rate of 44.2%, with reduced LVEF being a crucial negative prognostic factor [61,62].

Indeed, it is reasonable to raise uncertainties about whether moderate AS that is well tolerated by a ventricle with normal systolic function is equally well tolerated by a failing ventricle [63]. In fact, patients affected by both conditions face increased afterload from two different mechanisms: heightened sympathetic activity characteristic of HF and the high transvalvular gradient associated with aortic valve disease [64]. In this setting, the concept of valvulo-arterial impedance, which includes systolic blood pressure and mean transaortic gradient divided by SV index, captures these combined factors, offering a comprehensive assessment of LV afterload [65]. Pharmacological management of HFrEF (e.g., beta-blockers, ACE-inhibitors, etc.) can reduce only the systemic arterial compliance component of the overall afterload, without any beneficial effect on valvular impedance [66]. Particularly, in patients with AS alongside preexisting LV dysfunction, the additional afterload from AS, may exacerbate LV systolic dysfunction leading to detrimental clinical impact [67]. This “double-loaded ventricle” well describes the consequence of AS and HFrEF coexistence, particularly in elderly patients with increased arterial stiffness and hypertension.

There are three primary questions that a clinician should address when confronted with such a scenario, as these questions can guide the choice of treatment:

Is the AS truly moderate? The key takeaway in this context is to fully leverage the new frontiers offered by multimodal imaging, as indicated previously [68].

Is the LV systolic dysfunction caused by AS, or vice versa? In some cases, it is clear that a patient already has HFrEF due to a known cause, either ischemic or non-ischemic, and later develops aortic valve disease. However, when this is not obvious, it can be challenging to determine if LV systolic dysfunction is due to AS or other factors. When both conditions are present, assessing their individual impact on LV function is difficult [69]. In such situations, it may be helpful to adopt an ‘ex adiuvantibus’ approach: treat the AS and observe if LV dysfunction improves or stabilizes. If no other cause for LV dysfunction is found besides moderate AS, a lower threshold for recommending surgical or transcatheter AVR should be considered [70].

Who is the patient under consideration? Both the characteristics of the valvular disease and the patient’s individual attributes help decide whether to observe or intervene. Key factors include the rate of AS progression, degree of valvular calcification, condition of the vascular system including coronary arteries, and presence of aortic regurgitation or dilation [21]. However, before considering these technical aspects, it is vital to assess the patient’s frailty, ensuring their biological age matches their chronological age and understanding any significant comorbidities like renal disease, lung disease, or dementia, which may contribute to their symptoms [15]. 

## 5. Therapy

Medical therapy: There is currently no medical treatment for moderate AS, despite numerous promising therapeutic targets.

The results of several studies, including the Scottish Aortic Stenosis and Lipid Lowering Trial (SALTIRE) [71], the Simvastatin and Ezetimibe in Aortic Stenosis (SEAS) trial [72], and ASTRONOMER [73], indicate that treatment with HMG-CoA reductase inhibitors (statins) does not halt the progression of calcific AS or induce its regression. Current therapies under evaluation for long-term benefits in AS patients include angiotensin receptor blockers, Ataciguat [74], dipeptidyl peptidase-4 inhibitors [75], thiazolidinediones [16], and Phytin [44]. The SALTIRE II trial [76] demonstrated that neither denosumab nor alendronic acid affected the progression of AVC in patients with calcific AS. Elevated lipoprotein (a) (Lp(a)) and oxidized phospholipids on apolipoprotein B-100 (OxPL-apoB) are associated with faster AS progression and the need for AVR, particularly in younger patients [16]. This suggests a rationale for testing Lp(a)-lowering therapies, such as mipomersen [77], which has shown to lower Lp(a) levels in mice and humans. The FOURIER trial found that inhibiting PCSK9 with evolocumab, on a background of statin therapy, lowered LDL cholesterol levels and reduced cardiovascular event risk. Higher Lp(a) levels, rather than Lp(a)-corrected LDL cholesterol levels, correlated significantly with higher AS event risk, including AVR. The greater reduction of Lp(a) levels with PCSK9 could explain the benefit in AS. Additionally, promising non-lipidic therapeutic targets focus on the NOTCH1 pathway, a genetic driver of calcific aortic valve disease. Modifiers of NOTCH1 protein expression lead to cadherin-11 (CDH11) production by aortic valve interstitial cells, causing AS in mice [78,79,80]. Genetic or pharmacologic targeting of CDH11 can prevent calcific aortic valve disease and AS in Notch1 mutant mice [81]. However, development of a monoclonal antibody to CDH11 was discontinued after a phase 2b clinical trial for rheumatoid arthritis failed to achieve its primary outcome.

### Non-Medical Therapy

Currently, no completed randomized clinical trials justify AVR in high-risk moderate AS patients, such as those with LV dysfunction. To address this, several randomized studies involving TAVR and moderate AS are ongoing [2]. Guillaume and colleagues, in a retrospective study including 262 patients with moderate AS and HFrEF, have demonstrated that patients who underwent AVR had an improved survival compared with a matched group of patients who did not. Interestingly, only TAVR was associated with a better survival, whereas surgical AVR was not [82]. Nevertheless, no completed randomized clinical trials are available to date to justify the AVR even in high-risk subgroup of moderate AS, such as those with LV dysfunction. Trying to fill this gap, few randomized studies involving TAVR and moderate AS have been started and are currently ongoing. The “Transcatheter Aortic Valve Replacement to UNload the Left Ventricle in Patients With ADvanced HF (TAVR UNLOAD)” trial [83] aims to recruit 300 patients with HF and moderate AS who will be randomized to TAVR using a balloon-expandable bio-prosthesis (Edwards SAPIEN 3 valve) versus optimal medical therapy. The “PROGRESS Trial: Management of Moderate Aortic Stenosis by Clinical Surveillance or TAVR” (ClinicalTrials.gov ID NCT04889872) will enroll 750 patients with moderate AS and symptoms randomized to TAVR with a balloon-expandable bio-prosthesis versus medical therapy. Finally, the “Evolut EXPAND TAVR II Pivotal Trial” (ClinicalTrials.gov: NCT05149755), the only trial using a self-expandable bio-prosthesis, will randomize 650 patients with symptomatic moderate AS to TAVR versus medical therapy. The TAVR UNLOAD was started in 2016 with a theoretical end in 2023, but had a very slow enrolment rate that moved forward the estimated study completion to 2025. The other two studies started recently, and are expected to complete in 2037 and 2034 for the PROGRESS trial and the EXPAND TAVR II, respectively. Despite the potential benefits of TAVR in patients with moderate AS, such as afterload reduction, there are risks like pacemaker implantation and paravalvular leak-induced regurgitation. No preliminary data are available yet from these studies. Table 3 shows the ongoing studies regarding treatment of patients with moderate AS.

**Figure 3:**
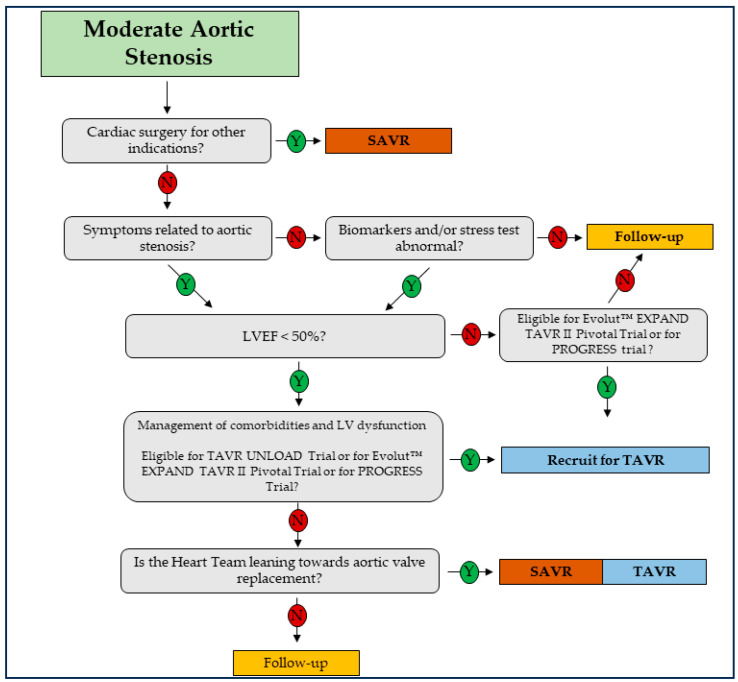
Proposed algorithm for managing patients with moderate aortic stenosis. SAVR: surgical aortic valve replacement; TAVR: transcatheter aortic valve replacement.

Figure 3 illustrates our proposed algorithm for managing patients with moderate AS. Specifically, for individuals who are not candidates for cardiac surgery due to other reasons, it is essential to use multimodality imaging to ascertain the presence of AS-related symptoms or significant biomarker abnormalities (e.g., NTproBNP).

## 6. Conclusions

Despite the advancements in non-invasive diagnostic technologies, challenges persist in the accurate diagnosis and interpretation of moderate AS. While the management of severe AS has undergone significant changes in recent years with the widespread use of TAVR, advancements in surgical techniques, and the identification of prognostic factors in asymptomatic patients enabling early intervention, the clinical strategy currently recommended to moderate AS remains a “watchful waiting”. This approach remains consistent regardless of the patient’s risk profile, the presence of LV dysfunction and symptoms. Despite that, many observational studies have reported substantial morbidity and mortality associated with moderate AS, particularly when associated with LV dysfunction. These findings suggest that the risk/benefit profile of AVR in moderate AS warrants re-evaluation and should be properly addressed through dedicated clinical trials, several of which are currently ongoing. While awaiting the results, a comprehensive and individualized approach on a case-by-case basis should be considered to ensure the best possible diagnostic and therapeutic management. In Table 4 we summarized clinical medical updates for aortic valve stenosis

## Figures and Tables

**Table 1:** Classification of the severity of aortic stenosis.

	Mild	Moderate	Severe
EOA	>1.5 cm^2^	1.0–1.5 cm^2^	<1.0 cm^2^
EOAi	>0.85 cm^2^/m^2^	0.6–0.85 cm^2^/m^2^	<0.6 cm^2^/m^2^
Gradient	<20 mmHg	20–40 mmHg	>40 mmHg
Velocity	<3.0 m/s	3.0–4.0 m/s	>4.0 m/s
DVI	>0.5	0.25–0.5	<0.25

EOA: effective orifice area; EOAi: indexed effective orifice area; DVI: Doppler velocity index.

**Table 2:** Six of the most representative studies of last decades regarding prognosis of patients with moderate aortic stenosis.

Study Name	Study Design	Study Population	Number of Patient	Primary End-Point	Result	Conclusion	Cit
Poor Long-Term Survival in PatientsWith Moderate Aortic Stenosis	Retrospective, multicentric	All patients with and without AS	241,303	All-cause and cardiovascular-related mortality	Increased risk of 5-year mortality: severe AS 3.0-fold and moderate 2.6-fold.	Moderate AS had a high risk of dying in the longer term that was similar to the risk in patients presenting with severe AS at baseline.	[9]
Mild and moderate aortic stenosis Natural history and risk stratification by echocardiography	Retrospective, single centre	Moderate and severe AS, normal EF	176	All-cause mortality	Survival of patients with mild and moderate AS was significantly worse than that predicted for age- and gender-matched control subjects, with an overall mortality that was 80% higher than that of the general population.	The presence of moderate to severe aortic valve calcification appears to be the most powerful predictor of outcome in mild and moderate AS and should, therefore, be determined in all patients	[52]
Natural History of Moderate Aortic Stenosis with Preserved and Low Ejection Fraction	Retrospective, single centre	Moderate AS with a propensity-matched cohort (1:3 ratio) without AS	952	All-cause mortality	Patients with moderate AS have increased mortality rates compared with control subjects in an unadjusted and adjusted analysis.	Patients with moderate AS have increased mortality rates in comparison with matched control subjects. This increased mortality is observed in patients with low or preserved EFs and even in patients with low transaortic gradients.	[8]
Outcomes of pseudo-severe aortic stenosis under conservative treatment	Retrospective, multicentric	Pseudo-severe low-flow/low-gradient AS defined with stress Echo Dobutamine	305	All-cause mortality		Pseudo-severe AS has a better survival rate than true-severe AS and comparable with that of propensity-matched patients with LV systolic dysfunction and no evidence of valve disease.	[53]
Prevalence and Prognostic implications of Discordant Grading and Flow-Gradient Patterns in Moderate Aortic Stenosis	Retrospective, multicentric	Moderate AS with concordant or discordant mean gradient	1974	All-cause mortality	Patients with discordant moderate AS showed significantly higher mortality when compared with patients with concordant moderate AS; on multivariable analysis, discordant moderate AS was independently associated with all-cause mortality.	Discordant grading is frequently observed in patients with moderate AS and is associated with increased risk of mortality compared with concordant moderate AS; among patients with discordant grading, the paradoxical and classical low-flow, low-gradient patterns but not the normal-flow, low-gradient pattern, are independently associated with worse outcomes.	[29]
Prognostic Implications of Moderate Aortic Stenosis in Patients With Left Ventricular Systolic Dysfunction	Retrospective, multicentric	Moderate and severe AS, reduced EF	305	All-cause death, AVR, and HF hospitalization at 4 year follow-up	All-cause death was 36%, HF hospitalization was 27%, and AVR occurred in 24% of patients.	Patients with concomitant moderate AS and LV systolic dysfunction are at high risk for clinical events.	[54]

AVR, aortic valve replacement; EF, ejection fraction.

**Table 3:** Ongoing studies regarding treatment of patients with moderate aortic stenosis.

Study Name	Device	Population	Study Design	Type of Study	Primary End-Point	Study Start	Study Completion (Estimated)	Cit
TAVR UNLOAD	Edwards SAPIEN 3 THV	Moderate AS	Randomized 1:1 Treatment vs. OHFT	International, multicenter, randomized, open-label	Non-hierarchical composite of all-cause death, disabling stroke, hospitalizations related to HF.	09-2016	02-2025	[11]
PROGRESS	Edwards SAPIEN 3/SAPIEN 3 Ultra/SAPIEN 3 Ultra RESILIA THV	Moderate AS	Randomized 1:1 Treatment vs. OHFT	International, multicenter, prospective, randomized, open-label	Non-hierarchical composite of death, and hf hospitalization or event.	12-10-2021	06-2037	ClinicalTrials.gov ID NCT04889872
EXPAND TAVR II	Medtronic Evolut PRO+ TAVR System, or Evolut FX TAVR System	Moderate AS	Randomized 1:1 Treatment vs. OHFT	International, multicenter, prospective, randomized, open-label	Composite rate of all-cause mortality, hf hospitalization or event, or medical instability leading to aortic valve replacement or re-intervention.	27-04-2022	12-2034	ClinicalTrials.gov ID NCT05149755

OHFT, optimal HF therapy; HF, heart failure.

**Table 4:** Clinical medical updates for aortic valve stenosis.

Clinical Medicine Updates for Aortic Valve Stenosis
Despite many observational studies have reported substantial morbidity and mortality associated with moderate AS, guidelines recommended a “watchful waiting” approach
Recent data indicates that around 40% of patients with moderate AS develop severe stenosis within approximately 2.5–3 years
Advancements in multimodal valve imaging technology have provided fresh insights into predicting disease progression and prognosis
AI algorithm capable of predicting progression rate from moderate to severe AS was developed
Prognostic value of LV remodeling patterns in moderate AS is associated with an increased risk of all-cause mortality
NT-proBNP concentrations are associated with clinical risk in the subsequent two years in individuals with non-severe AS
The risk/benefit profile of AVR in moderate AS warrants re-evaluation and should be properly addressed through dedicated clinical trials, several of which are currently ongoing
A shift from “standardization” to “individualization” in defining the severity of AS and management strategy is necessary to improve clinical outcomes and quality of life in patients with AS
Early interventional approach for moderate AS should be considered in some scenarios

AS, aortic stenosis; AI, artificial intelligence; LV, left ventricular; NT-proBNP, N-terminal prohormone of brain natriuretic peptide; AVR, aortic valve replacement.
